# Determinants of perceived usefulness, satisfaction and behavioral intention of using AI in lesson planning among English teachers

**DOI:** 10.3389/fpsyg.2026.1732508

**Published:** 2026-03-04

**Authors:** Qihua Sun, Fangzhou Jin, Liangyong Li

**Affiliations:** 1 School of Humanities, Lishui University, Lishui, Zhejiang, China; 2 The University of Hong Kong, Hong Kong, Hong Kong SAR, China; 3 Academic Affairs Office, Lishui University, Lishui, Zhejiang, China

**Keywords:** artificial intelligence, behavioral intention, English teachers, lesson planning, needs satisfaction, technology acceptance

## Abstract

Artificial Intelligence (AI) can help teachers plan lessons more efficiently, but it also raises concerns about increased cognitive load, loss of autonomy, and uniform lesson plans. This study aims to investigate drivers of English teacher perceived usefulness (PU), needs satisfaction (NS), and behavioral intention (BI) towards AI-assisted lesson planning tools. By integrating Technology Acceptance Model 2 (TAM2), Decomposed Technology Acceptance Model (DTAM) and Self-Determination Theory (SDT), we propose a research model positioning output quality (OQ), job relevance (JR), and result demonstrability (RD) as antecedents, PU and NS as mediators, and BI as the outcome variable. Data were collected from 485 English teachers via a questionnaire survey and data were analyzed using partial least squares structural equation modeling (PLS-SEM). The results revealed that OQ significantly enhances both PU and NS (*p* < 0.001). JR and RD significantly and positively influence PU (*β* = 0.435, *p* < 0.001 for RD; *β* = 0.185, *p* < 0.001 for JR) but show no significant direct effect on NS (*p* > 0.05). Furthermore, both PU (*β* = 0.428, *p* < 0.001) and NS (*β* = 0.180, *p* < 0.001) directly and significantly predict BI, with NS serving as a significant mediator in the PU-BI pathway (*β* = 0.095, *p* < 0.05). These findings offer a solid theoretical and empirical foundation for understanding the cognitive and psychological mechanisms underlying teachers’ AI adoption behavior, and provide targeted practical implications for the design and promotion of AI educational tools.

## 1 Introduction

Artificial intelligence (AI), particularly generative AI, is transforming education, as it is many other sectors. Generative AI tools such as ChatGPT, Doubao, Kimichat, and DeepSeek are seeing growing adoption in educational contexts globally, and this trend is especially pronounced in China, where educational digitalization and intelligent transformation have become national strategic priorities for foreign language education. In China’s English as foreign language (EFL) teaching landscape, the curriculum design and instructional practice are guided by the Core Competencies Framework articulated in the national English curriculum standards (the 2017 Edition with 2020 Revisions for senior high schools and the 2022 Edition for compulsory education). This core framework centers on four interwoven dimensions: language ability, learning ability, cultural awareness, and thinking capacity, and it sets stringent requirements for English teachers to design lesson plans that integrate disciplinary literacy cultivation, student-centered learning, and contextualized language application. Against this backdrop, English teachers across all educational stages in China are faced with the dual task of aligning daily teaching practice with national curriculum norms and meeting the diverse learning needs of students, making high-quality and targeted lesson planning a core professional requirement.

These AI technologies have provided teachers with unprecedented instructional support tools for their daily work (Ng et al., 2023). In that same daily practice, lesson planning (LP) stands out as one of the most time-consuming and core tasks. It serves as both a foundational component of instructional design and a key process whereby teachers translate pedagogical concepts into classroom practice. High-quality LP helps teachers clarify teaching objectives, optimize teaching content, and design effective teaching activities, thereby better meeting students’ learning needs (Shulman, 1987).

Nowadays, AI technologies are driving LP’s evolution from experience-based practice to data-informed, intelligent design (Zhang, 2025). This intelligent shift has become a crucial pathway to enhancing both the efficiency and quality of teaching. Existing studies show that AI boosts instructional effectiveness by supporting the creation of digital resources, identifying student learning styles, and fostering diverse pedagogical approaches (Zhang and Zhang, 2024). AI-assisted planning tools help teachers rapidly generate teaching content and activity designs tailored to instructional goals and student profiles. These tools deliver personalized resources, intelligent design suggestions, rich language materials, and automated evaluation, significantly broadening the possibilities for teaching and learning (Chounta et al., 2022). In language education specifically, AI supports developing lesson ideas, curating and translating instructional materials, and designing assessment tools (Napal Fraile and Badiola, 2024), in turn raising LP efficiency and helping teachers better address students’ individualized learning needs (Rudolph et al., 2023). For Chinese English teachers in particular, such tools offer potential solutions to the time and cognitive burden of designing lesson plans that adhere to the national Core Competencies Framework, while also enriching instructional content with diverse cultural and linguistic resources.

However, how teachers can effectively utilize AI tools for lesson planning remains a practical challenge. For instance, English language teachers face multiple hurdles in AI adoption, including stringent demands for linguistic accuracy and cultural sensitivity, as well as concerns about whether AI tools align with subject-specific pedagogical approaches. In the Chinese FLT context, these hurdles are further compounded by the imperative to ensure AI-generated lesson plans align with the national curriculum’s core competency goals and adapt to Chinese students’ actual learning levels—an issue that often forces English teachers to invest extra time in reviewing and revising AI-generated content, which may undermine their acceptance and use of such technology (Lee and Park, 2023). As such, the practical implementation of AI in lesson planning remains constrained by teachers’ specific needs and widespread adoption occurs only when the technology genuinely addresses those requirements. Ultimately, teachers’ intention to use AI tools is a key factor in determining the extent to which these technologies are successfully integrated into educational practice (Scherer et al., 2019; Teo, 2011).

To investigate the factors influencing English teachers’ intention to use AI in lesson planning, this study develops and validates a research model based on the Technology Acceptance Model 2 (TAM2) and the model incorporates three external variables, including output quality (OQ), job relevance (JR), and result demonstrability (RD) as predictors of perceived usefulness (PU) (Venkatesh and Davis, 2000), and introduces needs satisfaction (NS) (Roca et al., 2006; Ryan and Deci, 2000) as an additional mediator between PU and behavioral intention (BI). This study aims to address the following core question: What factors significantly influence English teachers’ behavioral intention when using AI-assisted lesson planning tools?

## 2 Literature review

### 2.1 Theoretical frameworks

**Figure 1:**
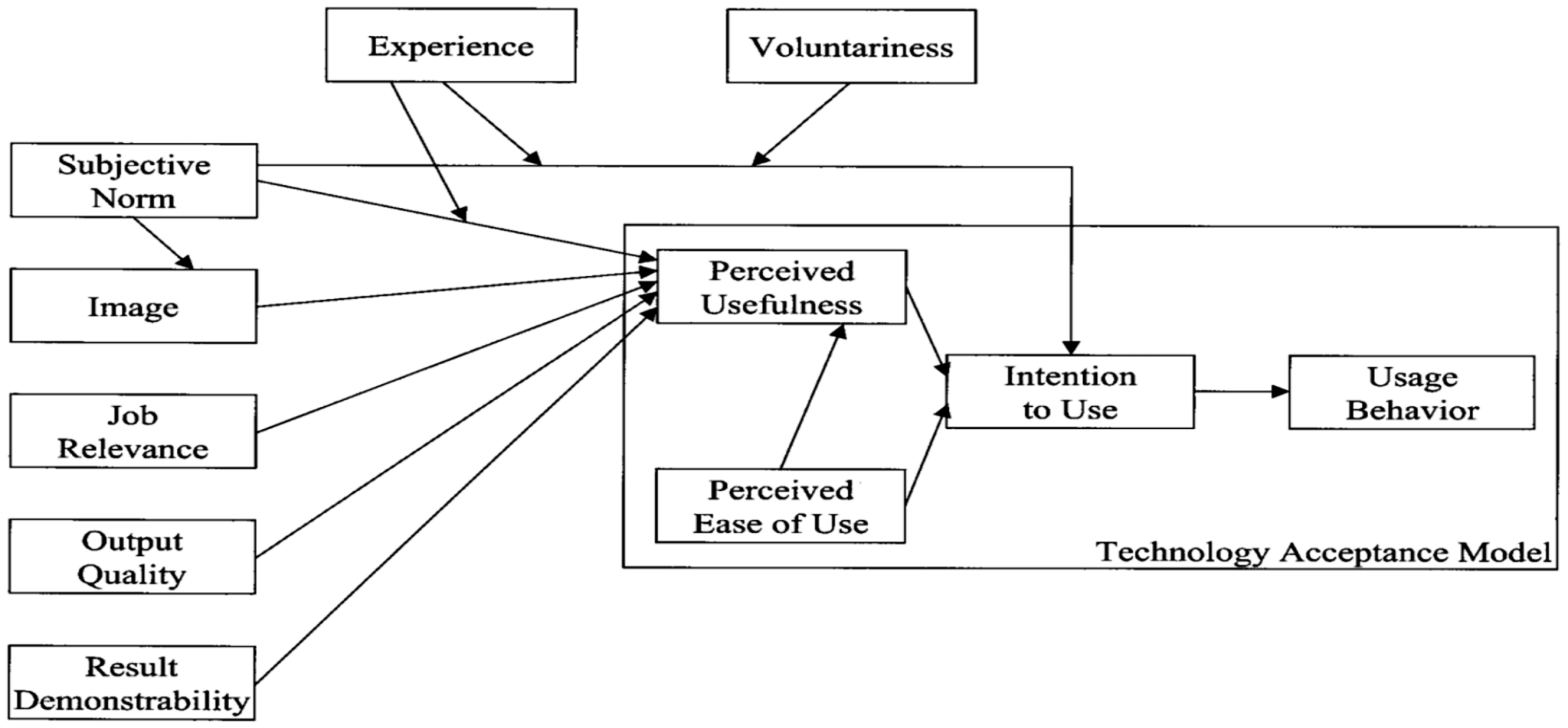
The TAM2 model.

This study employs the Technology Acceptance Model 2 (TAM2) (Venkatesh and Davis, 2000) (Figure 1) as its primary theoretical foundation, and it focuses specifically on cognitive instrumental processes to explain English teachers’ BI to adopt AI for LP. TAM2 extends the original TAM by incorporating social influence and cognitive instrumental processes, retaining PU and perceived ease of use (PEU) as core constructs while removing attitude as a mediating variable. Within this TAM 2 framework, OQ, JR, and RD are conceptualized as cognitive instrumental antecedents of PU. Empirical evidence confirms the model’s broad validation across contexts including e-commerce (Paramaeswari and Sarno, 2020), mobile applications (Khoa et al., 2021), and educational technology adoption (Altawalbeh, 2023; Virdi and Mer, 2023), which supports its applicability to this study.

To further contextualize teachers’ intention to use AI for lesson planning, this study also draws on the Decomposed Technology Acceptance Model (DTAM) (Roca et al., 2006) and Self-Determination Theory (SDT) (Ryan and Deci, 2000). DTAM integrates elements of Theory of Planned Behavior (TPB) (Ajzen, 1991), Technology Acceptance Model (TAM) (Davis, 1989), and Expectancy Disconfirmation Theory (EDT) (Oliver, 1980), and positions user satisfaction as a central predictor of continuance intention. According to DATM, sustained usage depends not only on PU but also on user satisfaction derived from factors including information quality, service quality, and cognitive absorption (Roca et al., 2006). SDT further enriches this theoretical perspective by emphasizing that satisfaction of psychological needs—autonomy, competence, and relatedness—fosters intrinsic motivation and sustained behavioral engagement with a technology (Ryan and Deci, 2000).

Building on these theoretical insights, this study introduces NS as a mediating variable linking cognitive evaluations (e.g., OQ, JR, RD), PU, and BI. It posits that English teachers’ adoption of AI-driven lesson planning is motivated by both instrumental utility and its ability to fulfill teachers’ deeper psychological and professional needs. Therefore, OQ, JR, and RD act not only as cognitive antecedents of PU within TAM2 framework but also as predictors to NS, thereby fostering sustained AI adoption intention through both cognitive and affective pathways (Hadji and Degoulet, 2016; Liu and Sutunyarak, 2024; Luo, 2024).

### 2.2 Hypotheses development and conceptual framework

In the Technology Acceptance Model 2 (TAM2), BI is positively influenced by two key factors: PU and PEU. PU refers to the degree to which an individual believes that using a particular technology or system would enhance their job performance. PU exerts a direct positive influence on BI, meaning that if users perceive a technology as useful, they are more likely to form an intention to use it (Venkatesh et al., 2003). Research has revealed that PU has a statistically significant direct effect on intention to use Web 2.0 technologies (Teo et al., 2019).

Moreover, the relationship between PU and user satisfaction has also received substantial empirical support. Recent studies demonstrate that PU significantly enhances user satisfaction with emerging technologies, particularly in the context of generative AI applications in educational settings. Zheng et al. (2024) found that PU positively influences pre-service teachers’ satisfaction with generative AI tools, while Han and Sa (2022) established that PU of online courses significantly contributes to educational satisfaction. In their study of ChatGPT usage in higher education, Yu et al. (2024) found that PEU and PU directly affect user satisfaction, which in turn positively influences continuance use intention.

Output quality, defined by Venkatesh and Davis (2000) as the perceived value and readiness of a system’s outputs for one’s professional use, has been widely recognized as a critical determinant of user satisfaction across various technology adoption contexts. Almufarreh (2024) identified content quality as a pivotal predictor of user satisfaction with generative AI tools in higher education, while Duong et al. (2024) found that both information and service quality significantly affect students’ satisfaction with ChatGPT for learning purposes. Ashfaq et al. (2020) further reinforced this relationship through their analysis of chatbot users.

Mateo et al. (2025) found that PEU, subjective norm, JR, OQ, and RD enhance the PU of the applications, which in turn influences users’ BI to adopt them. Additionally, factors such as subjective norms, professional reputation, JR, and OQ exert an indirect influence on intention, with PU serving as a key mediating variable (Garcia, 2024). Yazdanpanahi et al. (2024) further confirmed that subjective norm, JR, OQ, and RD have a significant positive influence PU. Furthermore, in their examination of EFL learners’ acceptance of ChatGPT, Hwang et al. (2025) found that RD has a significant positive predictor of PU.

Job relevance, defined as the perceived fit between AI tools and core instructional tasks (Venkatesh and Davis, 2000), directly fosters both competence and autonomy needs satisfaction. When AI applications are well aligned with teaching activities (e.g., lesson planning, resource customization), educators gain efficacy in achieving pedagogical goals and experience a greater sense of agency in integrating technology free from external pressure, thereby enhancing overall NS (Ryan and Deci, 2000). This finding is corroborated by Roca et al. (2006), who found that relevance exerts a positive influence on user satisfaction with e-learning systems.

Result demonstrability, defined as the clarity and communicability of the immediate value a system delivers (Venkatesh and Davis, 2000), also contributes to NS by reinforcing competence and autonomy. The positive effect of RD user satisfaction is not limited to education sector; similar outcomes have been observed in other fields, such as architecture (Algassim et al., 2025).

From the perspective of Self-Determination Theory (SDT), NS is underpinned by the fulfillment of three basic psychological needs: autonomy, competence, and relatedness (Deci and Ryan, 1985). BI represents a user’s willingness and behavioral tendency to adopt a certain technology in future practice (Ajzen, 1991; Davis, 1989). Prior research has yielded mixed yet insightful findings regarding the relationship between user satisfaction and BI in technology-mediated learning contexts. Liaw (2008) found that perceived user satisfaction exerts a significant positive effect on learners’ BI to adopt e-learning systems. Consistent positive effects of user satisfaction on BI have also been demonstrated across diverse contexts, including online professional learning communities (Jin et al., 2024), Massive Open Online Courses (MOOCs) (Pozón-López et al., 2021), electronic banking services (Khatoon et al., 2020), and service industries (Tuncer et al., 2021). In the specific context of AI tools, Yu et al. (2024) founded that user satisfaction significantly predicts continuance use intention for ChatGPT in higher education. Rekha et al. (2023) further confirmed that user satisfaction, alongside PU and computer self-efficacy, directly influences learners’ continuance intention in MOOC contexts. Wang et al. (2024) additionally demonstrated that both user satisfaction and task-technology fit both exert significant positive influences on continuance intentions toward educational video platforms.

This relationship, however, is more complex in the educator context. Within the teaching profession, NS derived from instructional and professional practice plays a crucial role in AI technology adoption. When teachers’ basic psychological needs are fulfilled, they are more inclined to demonstrate openness to sustained use of AI technologies. Despite the centrality of this construct, few empirical studies have examined the role of teachers’ NS in their acceptance and adoption of AI tools within educational contexts.

Based on prior theories and literature above, the following hypotheses are proposed as followed:

*H1*: OQ is expected to have a positive direct influence on PU.

*H2*: JR is expected to have a positive direct influence on PU.

*H3*: RD is expected to have a positive direct influence on PU.

*H4*: PU is expected to have a positive direct influence on BI.

*H5*: OQ is expected to have a positive direct influence on NS.

*H6*: JR is expected to have a positive direct influence on NS.

*H7*: RD is expected to have a positive direct influence on NS.

*H8*: PU is expected to have a positive direct influence on NS.

*H9*: NS is expected to have a positive direct influence on BI.

**Figure 2:**
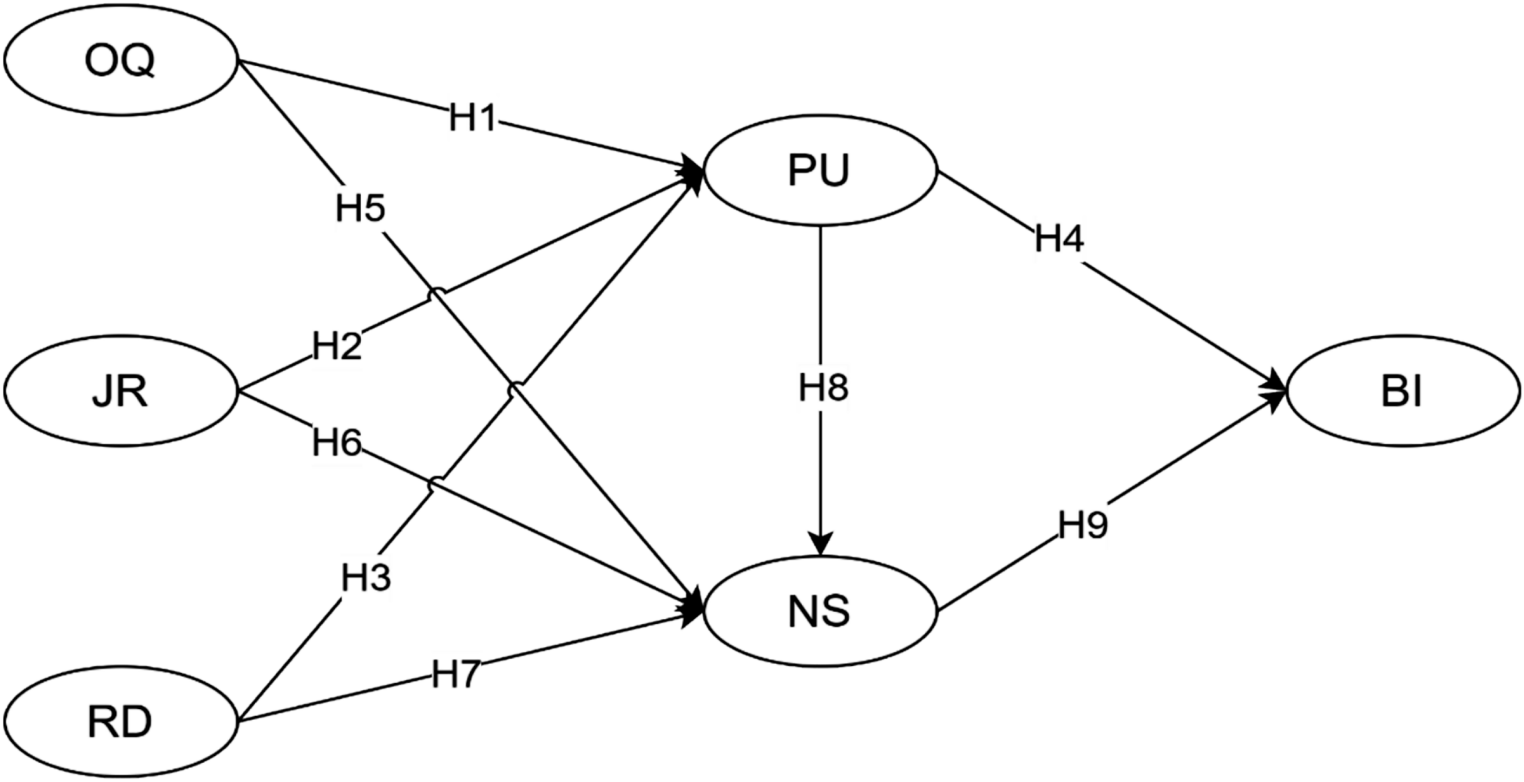
The conceptual framework. OQ, output quality; JR, job relevance; RD, result demonstrability; PU, perceived usefulness; NS, needs satisfaction; BI, behavioral intention.

Based on the theoretical foundations and research hypotheses developed above, we develop an integrated conceptual framework (Figure 2). This model proposes that three external variables, namely OQ, JR, and RD, serve as antecedents influencing both PU and NS. Additionally, PU influences NS. In turn, both PU and NS jointly explain English teachers’ BI toward AI-based lesson planning. The proposed model offers a comprehensive structure for testing the hypothesized relationships and highlights the roles of both technological perceptions and psychological fulfillment in the context of educational AI integration.

## 3 Methodology

This study adopts a partial least squares structural equation modeling (PLS-SEM) approach to develop a research model that captures the relationships among the six focal variables in the study: OQ, JR, RD, PU, NS, and BI. Data were collected via a questionnaire survey comprising demographic items and multiple-items scales for each variable in the research model.

### 3.1 Participants

Table 1 presents the demographic characteristics of the participating teachers. The total sample comprised 485 participants, including 306 secondary school English teachers and 179 higher education English teachers. For secondary school teachers, the cohort was predominantly female (86.3%). In terms of educational attainment, 84.3% held a bachelor’s degree and 12.4% possessed a postgraduate qualification. Regarding teaching experience, those with 6–15 years (30.1%) and 16–25 years (34.0%) of experience together made up 64.1% of this subgroup—over half the total. Additionally, 55.2% held a Level 1 professional title. These teachers were employed at regular middle schools (184, 60.1%) and key middle schools (122, 39.9%), and taught at the junior high (185, 60.5%) and senior high (121, 39.5%) levels.

**Table 1:** Demographic profile of the participants.

Demographic variables		Frequency	Percentage
Secondary school English teachers			
Gender	Male	42	13.7
	Female	264	86.3
Education level	Below bachelor’s degree	10	3.3
	Bachelor’s degree	258	84.3
	Postgraduate (master’s, doctoral)	38	12.4
Teaching experience	1–5 years	36	11.8
	6–15 years	92	30.1
	16–25 years	104	34
	Above 25 years	74	24.2
Professional title	Below level 1 teacher	67	21.9
	Level 1 teacher	169	55.2
	Senior teacher	68	22.2
	Professor-level senior teacher	2	0.7
Type of school	Regular middle school	184	60.1
	Key middle school	122	39.9
School level	Junior high school	185	60.5
	Senior high school	121	39.5
Higher education institutional English teachers			
Gender	Male	46	25.7
	Female	133	74.3
Education level	Bachelor’s degree and below	29	16.2
	Master’s degree	114	63.7
	Doctoral degree	36	20.1
Teaching experience	1–5 years	20	11.2
	6–15 years	32	17.9
	16–25 years	89	49.7
	Above 25 years	38	21.2
Professional title	Teaching assistant	14	7.8
	Lecturer	88	49.2
	Associate professor	66	36.9
	Professor	11	6.1
Courses taught	College English	75	41.9
	English major courses	79	44.1
	Other English courses	25	14

For higher education English instructors, females constituted 74.3% of the subgroup. In terms of academic qualifications, 63.7% held a master’s degree and 20.1% a doctoral degree. Nearly half (49.7%) reported 16–25 years of teaching experience, and faculty ranks included Lecturers (49.2%), Associate Professors (36.9%), and other positions (13.9%). In terms of teaching courses, the number of general English teachers (41.9%) and English major teachers (44.1%) was nearly equivalent, with teachers of other English disciplines accounting for the remaining 14.0%.

### 3.2 Instrument

The questionnaire comprised two sections. The first section collected demographic information, including gender, educational attainment, teaching experience, and professional title. Additional context-specific items were included for university and secondary school teachers respectively: university teachers provided information on the courses they taught, while secondary school teachers reported their school type and school level. The second section assessed teachers’ perceptions of OQ, JR, RD, PU, NS, and BI. The survey adopted a 5-point Likert scale, ranging from 1 (strongly disagree) to 5 (strongly agree), and all six constructs on the scale were adapted from prior empirical research. Table 2 presents the sample items for each construct and their respective original sources.

**Table 2:** The sample items of each construct.

Constructs	Sample items	Number of items	Adapted from
OQ	The quality of AI-assisted lesson planning output is high.	3	Venkatesh and Bala (2008)
JR	In my English lesson planning, using AI is important. In my English lesson planning, using AI is relevant.	3	Venkatesh and Davis (2000) Venkatesh and Bala (2008)
RD	I have no difficulty telling others about the results of using AI for lesson planning. The results of using AI for lesson planning are apparent to me.	4	Venkatesh and Davis (2000) Venkatesh and Bala (2008)
PU	Using AI improves my English lesson planning performance. Using AI increases my productivity in English lesson planning.	4	Venkatesh and Davis (2000) Teo (2011) Li et al. (2019)
NS	I am satisfied with the performance of using the AI for lesson planning.	3	Roca et al. (2006) Roca and Gagné (2008)
BI	I will continue to acquire information about AI-assisted lesson planning. I intend to use AI in my future lesson planning. I will recommend other English teachers to use AI for lesson planning.	5	Chai et al. (2021) Venkatesh et al. (2003) Chatterjee and Bhattacharjee (2020)

### 3.3 Data collection

Data were collected through an online survey completed voluntarily by middle school and university English teachers in China. The online questionnaire link was distributed to participants through snowball sampling via WeChat. The study was conducted in accordance with the ethical guidelines approved by the Institutional Research Ethics Committee (Approval No. LWRW2025003). Informed consent was obtained online from all participants before data collection. At the outset of the questionnaire, all respondents were informed of the study’s aim, and assured of their anonymity. A total of 522 responses were initially collected with 37 excluded from the subsequent analyses due to excessively short completion time or identical responses across all items, indicating potential careless responding. Following data cleaning, the final analytical sample comprised of 485 teachers, including 179 university teachers and 306 secondary school teachers.

### 3.4 Data analysis

In this study, data analysis was performed using partial least squares structural equation modeling (PLS-SEM), a robust approach suited to exploratory and predictive research focused on testing complex relationships among the study’s core variables: OQ, JR, RD, PU, NS, and BI (Hair Jr et al., 2017). PLS-SEM was selected for three key reasons: its flexibility in accommodating both reflective and formative constructs, its suitability for model development in applied research contexts, and its capacity to analyze data with smaller sample sizes compared to covariance-based SEM (Henseler et al., 2015). Analyses followed a two-step procedure: assessment of the measurement model (including validity, reliability and model fit) and evaluation of the structural model (focused on hypothesis testing).

## 4 Results

### 4.1 Descriptive statistics

Table 3 presents the descriptive statistics for the constructs measured in the study. The mean scores of all constructs exceeded the theoretical midpoint of 3, suggesting generally positive perceptions of the focal variables among participating teachers. BI yielded the highest mean score (*M* = 4.16, SD = 0.72), reflecting a strong willingness to adopt AI for lesson planning. This was followed closely by JR (*M* = 3.90, SD = 0.87) and PU (*M* = 3.89, SD = 0.72). OQ (*M* = 3.84, SD = 0.75) and NS (*M* = 3.76, SD = 0.73) also received favorable ratings, while RD had a comparatively lower, yet still positive, mean score (*M* = 3.65, SD = 0.79). Standard deviations ranged from 0.72 to 0.87, suggesting responses were reasonably concentrated across the constructs.

**Table 3:** Descriptive statistics of the study constructs.

Construct	*N*	Item	Mean	SD
OQ	485	3	3.8412	0.74735
RD	485	4	3.6495	0.78505
JR	485	3	3.8997	0.87487
PU	485	4	3.8851	0.71568
NS	485	3	3.7553	0.73022
BI	485	5	4.1621	0.72309

### 4.2 Testing the measurement model

**Figure 3:**
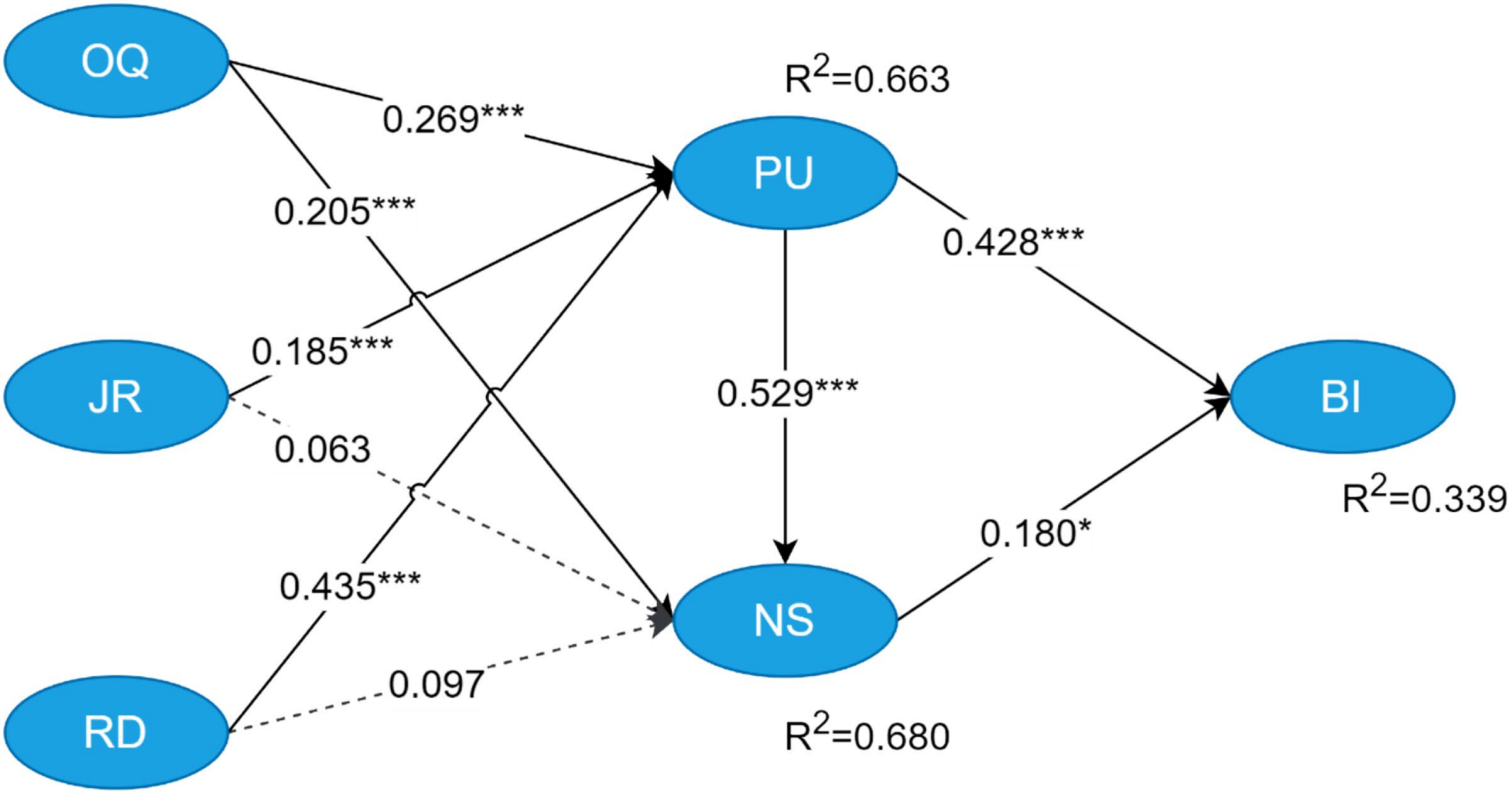
Path coefficients of the research model.

We conducted a comprehensive reliability and validity evaluation of the measurement model in accordance with established methodological guidelines (Hair et al., 2019). For reliability assessment, composite reliability (CR) and Cronbach’s alpha were calculated, with all constructs meeting the recommended thresholds: CR values ranged from 0.948 to 0.969 (exceeding the 0.7 benchmark), and Cronbach’s alpha values fell between 0.931 and 0.953 (also surpassing 0.7), indicating strong internal consistency (Chin, 1998; Fornell and Larcker, 1981). Convergent validity was confirmed using two key indicators: (1) all item outer loadings, which ranged from 0.736 to 0.969 and exceeded the recommended threshold of 0.7 (Hair Jr et al., 2017), and (2) average variance extracted (AVE) values for each construct, which ranged from 0.787 to 0.911 and all surpassed the 0.5 cutoff (Fornell and Larcker, 1981) (Table 4). Discriminant validity was assessed via the Fornell-Larcker criterion, which stipulates that the square root of a construct’s AVE must exceed its correlation coefficients with all other constructs (Henseler et al., 2015). For example, the square root of its AVE for JR is 0.955, and its correlation coefficients with other constructs (e.g., 0.750 with BI and 0.641 with NS) were all lower than this value (Table 5). Thus, the measurement model satisfied the Fornell-Larcker criterion for discriminant validity. Collectively, these results confirms that the measurement model exhibits satisfactory reliability and validity, justifying its use in subsequent structural model analyses. Additionally, collinearity among latent variables in the structural model was assessed using variance inflation factors (VIF). VIF values ranged from 2.441 to 3.445, all below the conservative threshold of 5.0, indicating no severe multicollinearity issues that could bias path coefficient estimates (Hair Jr et al., 2017) (Table 4). Finally, prior to structural model analysis, the overall model fit was evaluated using two key indices: the Standardized Root Mean Square Residual (SRMR) and the Normed Fit Index (NFI). The estimated model yielded an SRMR value of 0.088. According to the conventional cutoff criteria proposed by Hu and Bentler (1999), an SRMR value close to 0.08 is considered indicative of a relatively good fit between the model and the observed data. The current SRMR of 0.088 falls just slightly above this threshold, suggesting an acceptable level of fit in terms of residual discrepancy. The model yielded an NFI of 0.897 (Figure 3). While a conventional cutoff of 0.90 is typically recommended for acceptable fit (Bentler and Bonett, 1980), social science and educational technology research has noted that NFI values between 0.85 and 0.90 are deemed reasonable for exploratory studies, particularly those with a theoretical foundation that test novel variable relationships (Henseler et al., 2016).

**Table 4:** Reliability, validity, and predictor collinearity diagnostics.

Constructs	Items	Loadings	Cronbach’s alpha	Composite reliability	AVE	Inner VIF values
OQ	OQ1	0.913^***^	0.934	0.957	0.882	3.445 (OQ → NS)
	OQ2	0.953^***^				
	OQ3	0.951^***^				
JR	JR1	0.954^***^	0.952	0.969	0.911	2.441 (JR → NS)
	JR2	0.969^***^				
	JR3	0.941^***^				
RD	RD1	0.918^***^	0.940	0.955	0.842	3.380 (RD → NS)
	RD2	0.934^***^				
	RD3	0.902^***^				
	RD4	0.916^***^				
PU	PU1	0.930^***^	0.953	0.965	0.875	2.773 (PU → BI)
	PU2	0.938^***^				
	PU3	0.948^***^				
	PU4	0.926^***^				
NS	NS1	0.938^***^	0.934	0.958	0.883	2.773 (NS → BI)
	NS2	0.952^***^				
	NS3	0.928^***^				
BI	BI1	0.736^***^	0.931	0.948	0.787	
	BI2	0.903^***^				
	BI3	0.932^***^				
	BI4	0.951^***^				
	BI5	0.899^***^				

* = *p* < 0.05; ** = *p* < 0.01; *** = *p* < 0.001.

**Table 5:** Discriminant validity for the measurement model (Fornell–Lacker criterion).

Constructs	BI	JR	NS	OQ	PU	RD
BI	0.887					
JR	0.750	0.955				
NS	0.522	0.641	0.940			
OQ	0.610	0.736	0.722	0.939		
PU	0.572	0.682	0.800	0.747	0.935	
RD	0.542	0.688	0.711	0.787	0.773	0.917

### 4.3 Testing the structural model

Having established the reliability and validity of the measurement model, we employed PLS-SEM to assess the structural model and examine the hypothesized relationships (Sarstedt et al., 2022). This analytical method is especially well-suited for complex models featuring latent variables, given its ability to handle non-normal data and optimize the explained variance of endogenous constructs (Hair and Alamer, 2022). The structural model evaluation focused on path coefficients, their significance levels evaluated through 5,000-bootstrap samples, and the coefficient of determination (R^2^) for endogenous variables, which indicates the proportion of variance explained by the model (Henseler et al., 2015). Additionally, we assessed the effect size (*f*^2^) to quantify the practical significance of exogenous variables on endogenous constructs and predictive relevance (*Q*^2^) using blindfolding to validate the model’s predictive capability (Hair et al., 2019). The results are below.

Table 6 presents the outcomes for the hypothesis test and Figure 3 shows path coefficients of the research model. The PLS-SEM analysis with 5,000 bootstrap samples confirmed seven of nine hypothesized paths were statistically significant and supported (*p* < 0.01). The results show that OQ, JR, and RD are all significant antecedents of PU, with RD exhibiting the strongest effect (*β* = 0.435, *p* < 0.001). Furthermore, PU significantly influences both BI (*β* = 0.428, *p* < 0.001) and NS (*β* = 0.529, *p* < 0.001), confirming its central role in the model. OQ also shows a significant positive effect on NS (*β* = 0.205, *p* < 0.001). However, the effects of JR and RD on NS were not statistically significant (*β* = 0.063, *p* = 0.162; *β* = 0.097, *p* = 0.062, respectively), leading to the rejection of H6 and H7. Finally, NS demonstrates a significant, though relatively modest, positive effect on BI (*β* = 0.180, *p* < 0.05).

**Table 6:** Hypotheses testing of the research model.

300	Path	*β*	*t*	*p*	Results	*f* ^2^	*Q* ^2^
H1	OQ → PU	0.269	4.725	0.000	Accepted	0.066	0.594(NS); 0.573(PU); 0.264(BI)
H2	JR → PU	0.185	4.023	0.000	Accepted	0.043	
H3	RD → PU	0.435	8.057	0.000	Accepted	0.199	
H4	PU → BI	0.428	5.990	0.000	Accepted	0.100	
H5	OQ → NS	0.205	3.788	0.000	Accepted	0.038	
H6	JR → NS	0.063	1.398	0.162	Not Accepted	0.005	
H7	RD → NS	0.097	1.865	0.062	Not Accepted	0.009	
H8	PU → NS	0.529	10.179	0.000	Accepted	0.294	
H9	NS → BI	0.180	2.349	0.019	Accepted	0.018	

The model demonstrated strong predictive power, with *Q*^2^ values of 0.573 for PU, 0.594 for NS, and 0.264 for BI, all exceeding the 0.02 threshold for predictive relevance (Hair et al., 2019).

The mediation analysis reveals significant indirect pathways through which the exogenous variables influence NS and BI. OQ, RD, and JR all exert significant positive indirect effects on NS, exclusively mediated by PU (*β* = 0.142, *p* < 0.001; *β* = 0.230, *p* < 0.001; *β* = 0.098, *p* < 0.001, respectively) (Table 7).

**Table 7:** Mediating effects of the research model.

Source	Destination	Indirect effects	Total indirect effect
OQ	NS	OQ → PU → NS:0.142^***^	0.142^***^
RD	NS	RD → PU → NS:0.230^***^	0.230^***^
JR	NS	JR → PU → NS:0.098^***^	0.098^***^
OQ	BI	OQ → NS → BI:0.037	0.178^***^
	BI	OQ → PU → BI:0.115^***^	
	BI	OQ → PU → NS → BI:0.026^*^	
RD	BI	RD → PU → BI:0.186^***^	0.245^***^
	BI	RD → NS → BI:0.017	
	BI	RD → PU → NS → BI:0.41^*^	
JR	BI	JR → PU → BI:0.079^**^	0.108^***^
	BI	JR → NS → BI:0.011	
	BI	JR → PU → NS → BI:0.018	
PU	BI	PU → NS → BI:0.095^*^	0.095^*^

* = *p* < 0.05; ** = *p* < 0.01; *** = *p* < 0.001.

All three external variables demonstrate (OQ, JR, RD) significant total indirect effects. OQ influences BI through three pathways: a strong direct mediation via PU (*β* = 0.115, *p* < 0.001), a weaker but significant chain mediation through PU → NS (*β* = 0.026, *p* < 0.05), and a non-significant path through NS alone, resulting in a substantial total indirect effect (*β* = 0.178, *p* < 0.001). Similarly, RD affects BI primarily through PU (*β* = 0.186, *p* < 0.001) with additional minor contributions from other pathways, yielding the strongest total indirect effect among all predictors (*β* = 0.245, *p* < 0.001). JR shows a more modest but still significant total indirect effect on BI (*β* = 0.108, *p* < 0.001), mainly driven by the PU pathway (*β* = 0.079, *p* < 0.01).

Notably, PU itself demonstrates a significant indirect effect on BI through NS (*β* = 0.095, *p* < 0.05), confirming the partial mediation role of needs satisfaction in the technology acceptance process.

## 5 Discussion

This study aimed to investigate the determinants of English teachers’ intention to adopt AI-based lesson planning tools by integrating the Technology Acceptance Model 2 (TAM2), Decomposed Technology Acceptance Model (DTAM) and Self-Determination Theory (SDT). Specifically, we examined the mediating mechanisms through which external variables (OQ, JR, RD) influence teachers’ NS and BI.

### 5.1 Output quality (OQ)

The findings confirm that OQ significantly enhances both PU and NS, supporting H1 and H5, respectively. This demonstrates that high-quality outputs from AI tools, such as accurate teaching materials and pedagogically appropriate content, directly enhance teachers’ perceptions of the technology’s practical utility while simultaneously fulfilling their core psychological needs. The significant positive effect of OQ on PU aligns with prior findings from prior studies, where OQ consistently emerges as a crucial antecedent of PU across various technological contexts (Garcia, 2024; Jin et al., 2025b; Yazdanpanahi et al., 2024). Meanwhile, the direct relationship between OQ and NS corroborates research emphasizing quality dimensions as a fundamental driver of user needs satisfaction (Al-Fraihat et al., 2020; Almufarreh, 2024).

From a theoretical perspective, these dual effects can be understood through an integrated lens of technology acceptance and psychological need fulfillment. High-quality AI outputs directly boost teachers’ sense of professional competence by providing reliable, professionally aligned materials that reduce cognitive load in LP, thus addressing both the instrumental and psychological dimensions of educational technology adoption. When AI tools generate high-quality, instructionally relevant outputs, teachers are more likely to perceive these tools as genuinely useful for enhancing their instructional effectiveness. This heightened perception of usefulness subsequently fulfills their psychological needs for competence and autonomy by enabling more efficient and effective lesson planning practices (Roca et al., 2006; Ryan and Deci, 2000).

### 5.2 Job relevance (JR)

The results indicate that JR has a significant positive effect on PU, supporting H2. This finding is consistent with prior technology acceptance literature (Jin et al., 2025a), which identifies JR as a critical cognitive instrumental factor shaping users’ assessments of a technology’s utility. When teachers perceive AI tools as closely aligned with their core tasks, such as curriculum design and teaching material preparation, they are more likely to recognize the practical value of these tools, thereby elevating their perceptions of usefulness.

In contrast to the proposed hypothesis, JR did not exert a significant direct effect on NS, leading to the rejection of H6. This finding contrasts with certain prior studies in technology acceptance literature such as the work by Roca et al. (2006), which identified JR as a positive predictor of user satisfaction in e-learning systems. This discrepancy can be explained through a cognitive-affective mediating pathway. While AI-generated content (e.g., lesson plans, activity designs) may be relevant to core lesson-planning tasks, two key practical issues often emerge. First, such content often fails to meet teachers’ personalized needs (e.g., failing to align with the learning characteristics of specific grade levels or teachers’ habitual teaching styles) (Walter, 2024). Second, redundant information in some AI-generated content increases teachers’ cognitive load during the material screening and revision materials, thereby reducing overall lesson-planning efficiency (Mehta et al., 2025). In this context, teachers do not experience NS merely because AI-generated content is relevant to lesson planning. Instead, they first need to verify the practical utility of such content, for example, whether it reduces the workload of manual lesson planning or improves in-class effectiveness by optimizing activity designs (Ke and Gong, 2025). This process clearly demonstrates that the JR of AI-generated content to lesson-planning tasks can only influence teachers’ NS indirectly, through the mediating role of PU.

### 5.3 Result demonstrability (RD)

Our findings reveal that RD has a strong, significant positive effect on PU, supporting H3. This robust relationship indicates that when AI tools deliver tangible, observable benefits, such as clear efficiency gains or visible improvements to teaching materials, teachers are far more likely to perceive these tools as genuinely useful for their instructional practice.

This result aligns closely with prior technology acceptance research. The significant positive effect of RD on PU corroborates findings across multiple educational technology contexts, where the tangibility of outcomes consistently enhances perceived utility (Mateo et al., 2025; Yazdanpanahi et al., 2024). The substantial effect size (*β* = 0.435) further underscores RD’s crucial role as a cognitive instrumental factor in shaping teachers’ assessments of the practical value of AI tools. However, RD showed no significant direct effect on NS, leading to the rejection of H7. This finding contrasts with the recent work by Duong et al. (2024), which highlighted RD’s significant role in enhancing perceived value. This divergence can be explained by the inherent conceptual differences between these two constructs. RD refers to the tangibility and observability of an AI tool’s output and its effectiveness in communication (Moore and Benbasat, 1991; Venkatesh and Bala, 2008), a construct often associated with immediate, surface-level performance gains. In contrast, NS refers to the degree to which using a technology fulfills an individual’s innate psychological needs for autonomy, competence, and relatedness (Ryan and Deci, 2000). While AI-assisted lesson planning tools can demonstrably save time and propose enhanced instructional strategies, thus increasing perceived usefulness, their ultimate pedagogical efficacy is often delayed and must be evaluated post-hoc through student feedback, assessment results, and observations of classroom engagement (Zheng et al., 2024). This temporal disconnect creates a disjuncture whereby the immediate, demonstrable outcomes of the tool (high RD) fail to provide teachers with instant, intuitive feedback on their sense of professional competence or their pedagogical autonomy in the teaching process. Consequently, the direct pathway from RD to NS is disrupted, as the tangible results generated during the lesson planning phase do not immediately translate into intrinsic psychological need fulfillment in actual instructional use.

This intriguing divergence suggests that while demonstrable outcomes strongly shape cognitive evaluations of usefulness, they do not directly translate to psychological need fulfillment. The core distinction lies in their conceptual domains: RD addresses external, observable performance benefits, whereas NS encompasses internal psychological needs for autonomy, competence, and relatedness (Ryan and Deci, 2000). The immediate practical benefits captured by RD may therefore influence NS primarily through their enhancement of PU, rather than via direct psychological pathways.

### 5.4 Perceived usefulness (PU)

The findings demonstrate that PU has a significant positive influence both NS and BI, providing strong support for H4 and H8. These results align with the core propositions of TAM2 (Venkatesh and Davis, 2000) and extend their applicability to the context of AI-assisted lesson planning for English teachers.

The substantial positive effect of PU on NS (*β* = 0.529) reinforces and extends previous research into the psychological mechanisms underlying educational technology adoption. This finding corroborates Zheng et al.’s (2024) observation that PU enhances pre-service teachers’ satisfaction with generative AI tools, and also supports Al-Fraihat et al.’s (2020) identification of PU as a significant determinant of user satisfaction in e-learning systems. The strong positive relationship indicates that when teachers recognize the practical value of AI tools in improving lesson planning efficiency and effectiveness, this cognitive appraisal directly contributes to the fulfillment of their psychological needs, particularly those for competence and autonomy.

Furthermore, the notable positive effect of PU on BI (*β* = 0.428) substantially reinforces the foundational TAM2 proposition that perceptions of usefulness directly drive technology usage intentions (Venkatesh et al., 2003). This relationship exhibits remarkable consistency across technological contexts, echoing Teo et al.’s (2019) findings on Web 2.0 technologies and confirming the robustness of advanced AI applications in educational settings. The exceptionally strong path coefficient underscores the paramount importance of PU in determining English teachers’ willingness to adopt AI tools for lesson planning.

### 5.5 Needs satisfaction (NS) and mediating analysis

The results confirm that NS exerts a significant positive direct effect on BI, thereby supporting H9. This aligns with a core proposition of SDT (Ryan and Deci, 2000), which posits that fulfilling individuals’ basic psychological needs, autonomy, competence, and relatedness enhances their intrinsic and extrinsic motivation to engage in a given a behavior. This finding is consistent with prior research on educational technology adoption (Ashfaq et al., 2020), confirming that when teachers’ psychological needs are fulfilled through AI tools, their intention to adopt such technologies is significantly strengthened.

Mediation analysis further reveals a more nuanced pattern of effects. While NS was influenced by several antecedents, OQ, RD, and JR through PU indirectly through PU, its mediating role was limited. Crucially, NS only served as a significant mediator in the PU → BI relationship.

This specific mediating pathway is strongly supported by SDT (Deci et al., 2017) and recent research in educational technology (Wang et al., 2024). It indicates that when teachers perceive AI tools as useful for enhancing lesson quality, this perception satisfies their underlying psychological needs for competence (by mastering an effective instructional tool) and autonomy (by providing flexible approaches to task completion). This user satisfaction then acts a key psychological mechanism that translates the cognitive judgment of usefulness into a stronger BI to adopt the technology.

However, the direct mediating paths from OQ, RD, and JR to BI via NS alone (i.e., OQ → NS → BI; RD → NS → BI; JR → NS → BI) were not statistically significant. This unexpected pattern can be explained by a potential “quality-threshold effect” (Kelchtermans, 2009). While factors such as information quality may meet teachers’ basic requirements for effective lesson planning, this baseline level of satisfaction may be insufficient on its own to directly drive adoption intention. Teachers may require additional, more compelling incentives, such as the promise of long-term efficiency gains or significant workload reduction, to fully convert psychological need satisfaction into a decisive behavioral intention to act.

## 6 Conclusion

This study yields several major findings through the integration of TAM2, DTAM and SDT. First, it confirms that both PU and NS are significant direct predictors of BI to adopt AI assisted-lesson planning tools. Second, it clarifies the distinct roles of external antecedent variables: OQ exerts a robust positive influence on both PU and NS, while JR and RD significantly enhance PU but fail to exert a direct effect on NS. Third, and most notably, mediating analysis uncovers a critical nuance: NS acts as a significant mediator specifically in the PU-BI pathway. This indicates that the fulfillment of psychological needs contributes as a key mechanism through which cognitive appraisals of a tool’s usefulness are translated into stronger usage intentions, with direct NS-mediated pathways from other focal variables to BI not reaching statistical significance.

The findings of this study have some implications. Theoretically, the study validates the value of integrating technology acceptance models with motivational theory, demonstrating that a comprehensive understanding of AI adoption in education requires consideration of both cognitive evaluations and intrinsic psychological needs. This is particularly meaningful in the context of China’s EFL teaching landscape, where teachers must balance national curriculum mandates (e.g., the Core Competencies Framework) with personalized instructional design. This research further refines theoretical boundaries by revealing that cognitive instrumental factors (i.e., JR and RD) directly shape perceptions of usefulness yet do not inherently fulfill psychological needs. This is particularly evident in the “pedagogical autonomy conflict” (Kelchtermans, 2009): when the preset logic of AI tools contradicts teachers’ personalized teaching needs, such as the requirement to align lesson plans with China’s Core Competencies Framework, even if the tool is highly relevant to their work and its effects are quantifiable, it may not alleviate teachers’ psychological discomfort. Moreover, the specific mediating role of NS in the PU → BI pathway advances the study’s theoretical model, framing NS as a crucial psychological bridge that transforms instrumental utility into motivational force of AI adoption.

In practice, to address the key barriers identified in this study and promote the practical application of AI lesson planning tools, targeted strategies should be implemented by both AI tool developers and school administrators. For AI developers, the imperative is to prioritize high-quality, accurate outputs that align with China’s Core Competencies Framework, and design features that not only deliver clear benefits for core instructional tasks but are also engineered to actively support teachers’ autonomy and competence. For instance, by providing customizable templates that incorporate the four dimensions of the Core Competencies Framework (language ability, learning ability, cultural awareness, and thinking capacity), tools can reduce the cognitive burden of aligning AI-generated content with national curriculum goals, while also empowering teachers to adapt suggestions to their students’ diverse learning needs. For school administrators, professional training programs must go beyond merely demonstrating a tool’s utility; instead, they should strategically emphasize how AI tools empower teachers to meet national curriculum standards more efficiently, boost their professional efficacy, and articulate the long-term value that can sustain adoption motivation beyond initial satisfaction. Training could, for example, include workshops on using AI tools to design lesson plans that integrate cultural awareness and thinking capacity, directly addressing the requirements of the Core Competencies Framework. For teachers themselves, these findings confirm that thoughtfully selected AI tools can serve a vehicle for enhancing professional effectiveness and job satisfaction, particularly in a context where they face the dual pressure of adhering to national curriculum norms and meeting diverse student needs. By leveraging AI to streamline the alignment of lesson plans with the Core Competencies Framework, teachers can free up time to focus on student-centered, contextualized instruction, thereby reducing their cognitive load and enhancing their sense of professional autonomy.

Based on the development and validation of the proposed model, this study has three limitations. First, while this study included English teachers from secondary schools and higher education institutions across central, eastern, and southern China, the overall sample size remained limited, which may constrain the statistical robustness and generalizability of the findings to other geographic contexts (Polit and Beck, 2010). Second, this study’s cross-sectional design restricts causal inference and the observation of temporal changes, as it fails to clarify the temporal sequence of variables to establish casual relationships. For instance, it cannot confirm whether teachers first perceive AI as useful prior to forming BI, or only gradually develop such perceptions of usefulness after forming BI. Additionally, this design cannot capture the dynamic evolution of variables; for example, it is unable to track changes in teachers’ NS when using AI for lesson planning across different semesters, which hinders an exploration of the long-term stability of variable relationships (Kline, 2023). Third, the exclusive focus on English teachers also warrants future research to verify the model’s applicability across diverse academic disciplines and cultural contexts.

Future research should replicate the model with teachers across different countries and educational levels to test the cross-contextual invariance of the framework (Milfont and Klein, 2018). This would clarify whether OQ, JR, and RD have consistent effects across diverse educational contexts. Additionally, given the limitations of the cross-sectional design, including its inability to capture the temporal dynamic relationships and the causal order of variables, as well as its challenges in ruling out reverse causality and third-variable interference, future studies should adopt a longitudinal tracking design. Finally, building on the study’s emphasis on teacher needs satisfaction, future research could disentangle the unique incremental effects of autonomy, competence, and relatedness on the key outcomes of the model.

## Data Availability

The original contributions presented in the study are included in the article/supplementary material, further inquiries can be directed to the corresponding author.
